# Molecular Approaches to Protein Dimerization: Opportunities for Supramolecular Chemistry

**DOI:** 10.3389/fchem.2022.829312

**Published:** 2022-02-08

**Authors:** Dung Thanh Dang

**Affiliations:** Faculty of Biotechnology, Ho Chi Minh City Open University, Ho Chi Minh City, Vietnam

**Keywords:** approaches, inducers, protein, dimerization, supramolecular chemistry

## Abstract

Protein dimerization plays a key role in many biological processes. Most cellular events such as enzyme activation, transcriptional cofactor recruitment, signal transduction, and even pathogenic pathways are significantly regulated via protein-protein interactions. Understanding and controlling the molecular mechanisms that regulate protein dimerization is crucial for biomedical applications. The limitations of engineered protein dimerization provide an opportunity for molecular chemistry to induce dimerization of protein in biological events. In this review, molecular control over dimerization of protein and activation in this respect are discussed. The well known molecule glue-based approaches to induced protein dimerization provide powerful tools to modulate the functionality of dimerized proteins and are shortly highlighted. Subsequently metal ion, nucleic acid and host-guest chemistry are brought forward as novel approaches for orthogonal control over dimerization of protein. The specific focus of the review will be on host-guest systems as novel, robust and versatile supramolecular approaches to modulate the dimerization of proteins, using functional proteins as model systems.

## Introduction

**FIGURE 1:**
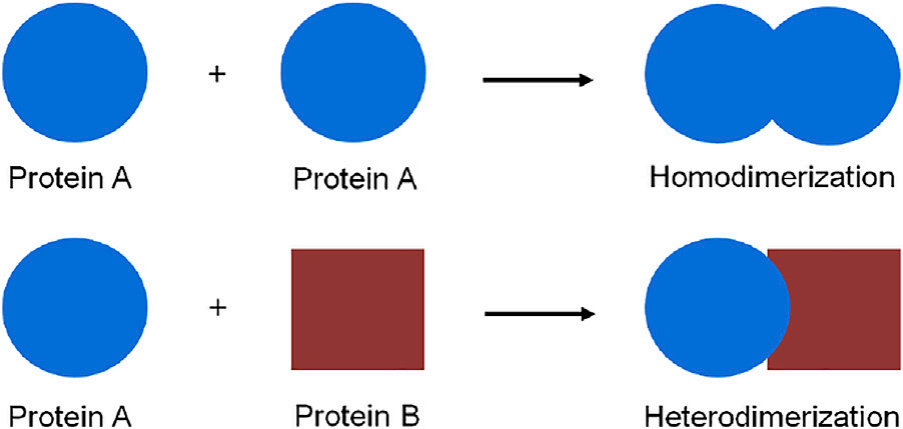
Schematic representation of protein homo-and hetero-dimerization.

Protein dimerization is a crucial biological process in which proteins interact, as for example homo- or hetero-dimers, to form a functional assembly (Figure 1). In fact, proteins rarely show function and activity in their isolated form in a biological environment. The self-assembly of proteins to form dimers or higher oligomeric aggregates is a common biophysical phenomenon, which occurs in every cellular compartment such as cell membranes, the nucleus, and the cytosol. All cellular pathways such as enzymatic activation (Citri and Yarden, 2006; Baselga and Swain, 2009), signal transduction (Ferrer-Soler et al., 2007; Ahsan, 2016), and even pathogenic pathways (Hynes and Lane, 2005) are significantly regulated *via* protein dimerization.

Regulation of protein dimerization is an essential process for the growth and development of organisms under the stimuli of intrinsic or extrinsic factors in the natural environment (Marianayagam et al., 2004). Therefore, understanding and modulating the molecular mechanisms of protein dimerization and their function represents the cutting edge of research and provides multiple entries for biomedical applications. Protein engineering represents one approach to induce or control protein dimerization, thereby facilitating an increase in protein stability and/or function (Grueninger et al., 2008; Ardejani et al., 2011). For example, engineering a dimeric interface of initiator caspase-9 resulted in caspase activation through induced proximity. Shi and coworkers have generated a dimeric caspase-9 by replacing five residues in the β6 strand of caspase-9 (Gly^402^-Cys-Phe-Asn-Phe^406^) with those normally present in caspase-3 (Cys^264^-Ile-Val-Ser-Met^268^), resulting in a dimeric interface of an engineered caspase-9. The engineered caspase-9 functioned as homodimer in buffered solution, leading to an increase in enzymatic activity *in vitro* and in cell-based studies (Chao et al., 2005). Additionally, protein can be engineered to feature enhanced dimerization via the introduction of coiled-coil zipper sequences (Junius et al., 1996; Mason and Arndt, 2004). The coiled-coil zipper functions through hydrophobic interactions between leucine rich motifs which form homo- or hetero-dimeric states. An example in this respect is the dimer formation between c-Jun and c-Fos, to form functional DNA transcriptional factors (Mason and Arndt, 2004; Gazon et al., 2017). The dimer formation of leucine-rich zippers provides a bioengineering approach that enables induced dimerization of proteins bearing leucine-rich repeats. The introduction of a leucine zipper motif to quiescent cell proline dipeptidase (QPP), enabled QPP homodimerization, which is essential for QPP activation (Chiravuri et al., 2000). The leucine zipper motifs were also applied to the induced dimerization of other proteins such as protein kinase (MLK-3) (Leung and Lassam, 1998) and tyrosine hydroxylase (Vrana et al., 1994) in which leucine zipper-induced protein dimerization showed a significant increase in enzymatic activity. Notwithstanding the great success achieved, the current approaches to engineered protein dimerization interfaces have their limitations, especially in terms of control over dimerization of protein dimerization event. Mutations made in the active domains of target proteins may change their biological structure and function. Approaches based on the addition of dimeric interfaces, such as *via* mutation interfaces or addition of leucine zipper-induced domains, do not provide a switching mechanism for temporal control or regulation of the dimerization. Therefore, the function of the resulting proteins in their biological processes is difficult to control. Protein dimerization approaches which are based on external molecular signals, capable of inducing or blocking dimerization, provide a strong point of entry to explore and control the molecular mechanisms of protein dimerization.

**FIGURE 2:**
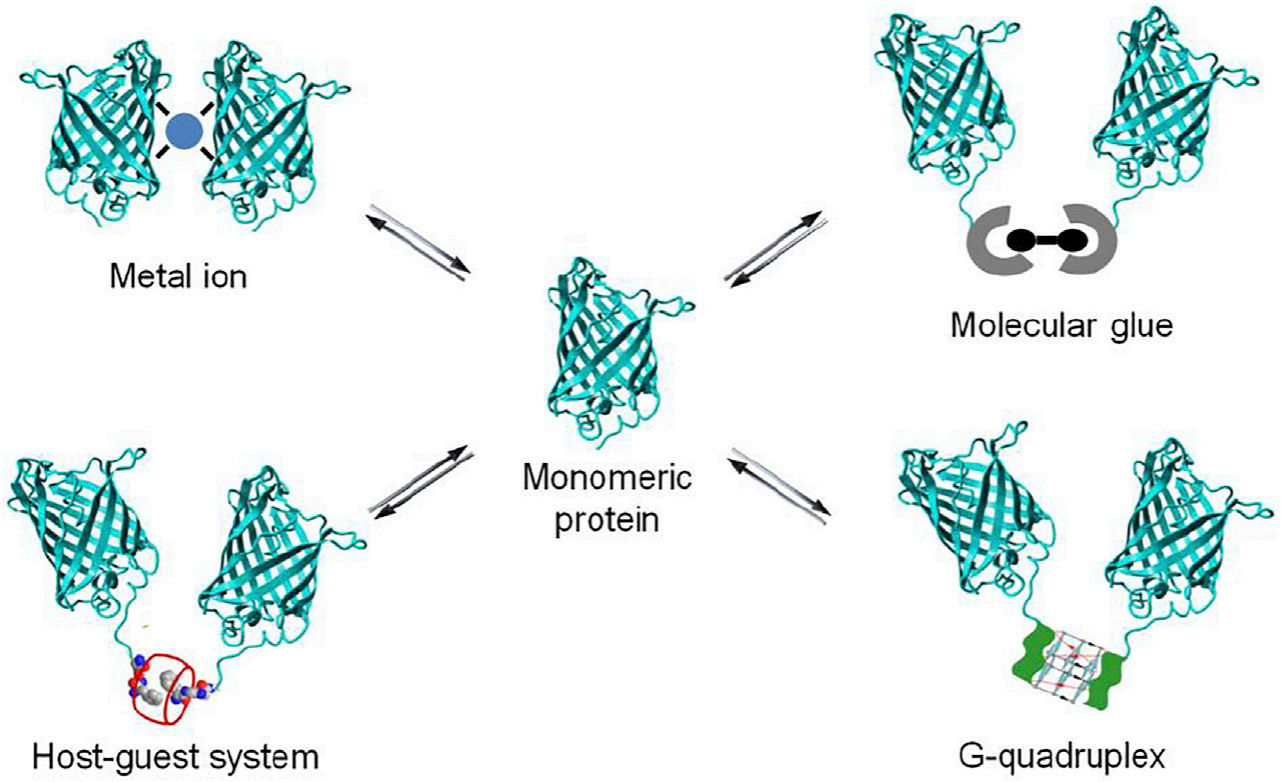
Schematic representation of dimerization of protein *via* molecular approaches.

A powerful chemical approach currently used to control protein dimerization is via the use of molecular glues. More recently as well the use of metal ion, nucleic acid and synthetic host-guest systems has been explored (Figure 2). All these chemical elements act by bringing two proteins together to induce dimerization, resulting in the activation or inhibition of biological events. The reversibility of chemically-induced protein dimerization is attractive for biomedical research, as it enables an added degree of control over protein dimerization and activation. In this review, a schematic overview and selected examples of protein dimerization mediated by different molecular inducers of dimerization are provided, with a focus on the supramolecular chemistry based approaches. Synthetic host-guest systems are brought forward as novel, robust and versatile entries to modulate the dimerization of proteins.

## Dimerization of Protein via Molecular Chemistry

### Molecular Glue-Induced Dimerization of Protein

**FIGURE 3:**
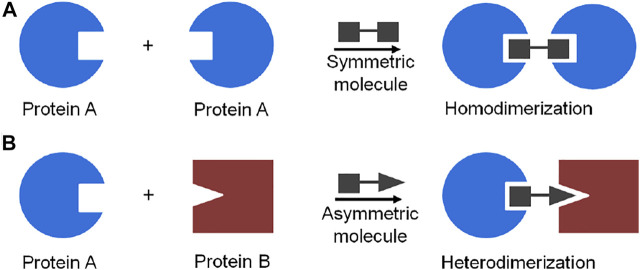
Schematic representation of molecule glue induced dimerization of protein: **(A)** protein homodimerization induced by a symmetric bifunctional molecule, **(B)** protein heterodimerization induced by an asymmetric bifunctional molecule.

A powerful chemical approach currently used to control protein dimerization is via the use of molecular glues (Schreiber, 2021). The concept of molecular glue-induced protein dimerization is based on the use of low molecular weight organic compounds bearing bifunctional moieties which interact simultaneously with two proteins or protein domains (Boyd et al., 2021). A chemical inducer of protein dimerization acts as a dimerizer to bring protein molecules together and form either a homo- or a heterodimer (Corson et al., 2008; Fegan et al., 2010; Boyd et al., 2021) (Figure 3).

Molecule glue approaches to induce protein dimerization have been demonstrated into two groups: 1) Asymmetric molecules such as Cyclosporin A (Liu et al., 1991), FK506 (Liu et al., 1991; Ho et al., 1996), FKCsA (Belshaw et al., 1996a), rapamycin (Rivera et al., 1996), gibberellin (Miyamoto et al., 2012), abscisic acid (Liang et al., 2011), HaXS (Erhart et al., 2013), TMP-HTag (Ballister et al., 2014) and ATB-737 (Hill et al., 2018) induce hetero-dimerization of proteins; 2) Symmetric molecules such as FK1012 (Spencer et al., 1993), coumermycin (Farrar et al., 1996) and (cyclosporin A)_2_ (Belshaw et al., 1996b) induce homo-dimerization of proteins (Table 1). For example, the natural product rapamycin has emerged as the biofunctional dimerizer to induce heterodimerization of proteins (Choi et al., 1996; Liang et al., 1999; Bayle et al., 2006; Brown et al., 2015; Mangal et al., 2018; Courtney et al., 2021). The most prominent molecular feature of rapamycin is its two chemically distinct protein binding domains: one part of the molecule binds with high nanomolar affinity to the FK506-binding protein (FKBP12), the other molecular part to the FRB domain of mTOR, FRAP (FKBP-rapamycin associated protein), overall resulting in dimerization of the proteins involved. Rapamycin is thus capable of inducing heterodimerization of fusion proteins featuring FKBP and FRB domains. In contrast to rapamycin, coumermycin has two of the same protein-binding moieties and can be used to induce homodimerization of GyrB (bacterial DNA gyrase B) (Farrar et al., 1996; Farrar et al., 2000; Cele et al., 2016; Broeck et al., 2019). The concept of molecule glue-induced protein dimerization can be extended to novel synthetic compounds as well. For example, a synthetic dimer of FK506, named FK1012, promotes FKBP12 homodimerization (Schultz and Clardy, 1998), or a synthetic dimer of cyclosporine named (CsA)_2_ can induce dimerization of cyclophilin (Belshaw et al., 1996b). These molecule glues are also capable of inducing protein dimerization in cases where the dimerizing protein of interest has been fused to a suitable protein ligand binding domain.

**TABLE 1:** Molecule glues-induced dimerization of proteins.

Molecule glues	Induced protein dimerization
Cyclosporin A Liu et al. (199)1	Cyclophilin-Calcineurin
FK506 Liu et al. (1991); Ho et al. (1996)	FKBP-Calcineurin
FKCsA Belshaw et al. (1996a)	FKBP-CyPFas
Rapamycin Rivera et al. (1996)	FKBP-FRB domain of mTOR
Gibberellin Miyamoto et al. (2012)	Gal-GID1
Abscisic acid Liang et al. (2011)	ABI-PYL
HaXS Erhart et al. (2013)	SNAPTag-HaloTag
TMP-HTag Ballister et al. (2014)	eDHFR-HaloTag
ATB-737 Hill et al. (2018)	Bclxl-Fab (AZ1)
FK1012 Spencer et al. (1993)	FKBP-FKBP
Coumermycin Farrar et al. (1996)	GyrB-GyrB
(Cyclosporin A)_2_ Belshaw et al. (1996b)	Cyclophilin- Cyclophilin

Protein dimerization induced by the specific binding of cell permeable high affinity small natural products or synthetic molecules represents a powerful tool for controlling dimerization of proteins in numerous biological processes such as gene expression (Rivera et al., 2012; Schreiber, 2021), proteolysis targeting chimera (PROTAC) (Mootz and Muir, 2002; Xu and Evans, 2005; Pratt et al., 2007; Foight et al., 2019), and signaling cascades (Shahi et al., 2012; Lecointre et al., 2018; Fujikawa et al., 2019). For example, control over gene expression was achieved with rapamycin by recruiting activation and repression protein domains to targeted loci (Schreiber, 2021). Fusion of an FRB domain to an activation domain (VP16) and a DNA-binding domain (Gal4) to an FKBP domain led to rapamycin-induced dimerization generating transcriptional activator functionality, and the promotion of gene expression (Liberles et al., 1997; Hardwick et al., 1999). Since toxicity of the natural rapamycin inhibits cell proliferation, Crabtree and co-workers have developed non-toxic rapamycin analogs which were successfully used to control gene expression (Bayle et al., 2006). The use of molecule glues induced protein dimerization to control the stability or rescue of proteins in living cells has also been demonstrated. Crabtree and coworkers described that FRB^*^ -not only bound to FKBP12 in the presence of a rapamycin analog (C20-MaRap) but also bore functionality which conferred reversible instability on the fusion proteins. In the absence of rapamycin analog, the glycogen synthase kinase-3β (GSK-3β) fused to FRB^*^ (GSK-3βFRB^*^) was rapidly degraded (Stankunas et al., 2003). Interestingly, C20-MaRap induced dimerization of FKBP12 and GSK-3βFRB^*^, which might lock FRB^*^ in a folded state, resulting in the stabilization of the GSK-3β protein. This system may provide a means to control the stability or degradation of target proteins. Another robust approach to rescue proteins from the proteasome is by using both molecule glue induced dimerization and splicing of ubiquitin hydrolysis. For example, split ubiquitin for the rescue of function (SURF) was based on the complementation of genetically split ubiquitin under the control of rapamycin-induced dimerization of FRB and FKBP. The strategy was as follows: 1) the FKBP was fused to the N-terminal fragment of ubiquitin to form FKBP-Ub^N^, 2) the C-terminal fragment of ubiquitin was fused to a protein of interest and FRB to form the FRB-Ub^C^-protein complex, and subsequently fused to a degradation signal (degron) which would cause degradation of the fusion protein by proteasome recognition. In the absence of rapamycin, the proteasome recognizes and “kills” the fusion protein through degron domain recognition, thereby promoting degradation of fusion protein. Addition of rapamycin caused dimerization of FRB and FKBP, which resulted in the reassembly and function of ubiquitin, thereby releasing the protein of interest from the degron and rescuing its function (Pratt et al., 2007). In addition, molecular glue-induced protein dimerization has also been investigated to control the activation of certain kinase family members, in order to study signal transduction (Belshaw et al., 1996b; Spencer et al., 1996; Kim et al., 2020). A synthetic molecule glue - FK1012 - induces homo-dimerization of FKBP and can also be used to gain control over programmed cell death. The Fas cytoplasmic domain was fused between poly FKBP and myristoyl group which located on the cell membrane. The presence of FK1012 mediated the aggregation of the Fas-poly FKBP receptor leading to activation of Fas signaling transduction, and eventually cell death (Spencer et al., 1996). An engineered rapamycin-induced dimerization approach of Fas consisting of FKBP and FRB proteins allowed rapamycin to specifically induce cellular apoptosis (Kim et al., 2020). Thus, the molecule glue approach is highly valuable for fundamental studies, drug development, and other biomedical applications. This approach, however, does require the construction of large fusion proteins, in which the required protein domains contribute substantial mass to the final protein construct, potentially affecting the biological activity of the target protein (Spencer et al., 1993; Fegan et al., 2010). Alternative methods for molecular control over protein dimerization are additionally required. Apart from molecular glue based approaches, metal ion, nucleic acid and host-guest chemistry are brought forward as novel approaches for orthogonal control over dimerization of protein.

### Metal Ion-Induced Dimerization of Protein

Metal ion-mediated protein dimerization has recently been demonstrated (Sinclair, 2012; Song et al., 2014; Kochanczyk et al., 2016). Tezcan and co-workers for example generated hybrid coordination motifs based on the simultaneous binding of a metal ion to a natural histidine amino acid and a non natural ligand on the α-helical surface of protein cytochrome cb_562_ (Radford et al., 2010). The ligand, 5-amino-8-hydroxyquinoline, which binds metal ions with high affinity, was covalently ligated to cysteine at position 70 of cytochrome cb_562_. Addition of metal ions such as Ni^2+^, Co^2+^, Cu^2+^ and Zn^2+^ induced cytochrome cb_562_ dimerization, resulting in an increase of global protein stability. Zn ions were also exploited as powerful metal ions to assemble protein in a homodimer (Brodin et al., 2010; Churchfield et al., 2016) and tetramer, in which four Zn ions associated at the surface of each protein monomer. The design and synthesis of a helical coiled-coil by metal-induced folding has also been demonstrated; fusing the Cys-X-X-Cys metal binding domain of rubredoxin to a target random coil peptide enabled Cd^2+^ to induce peptide dimerization (Kharenko and Ogawa, 2004). Interestingly, metal ion-induced protein dimerization was used to generate a structural superposition closely resembling bZip-type transcriptional factors, suggesting potential applications for the recognition of biological targets. Using metal ions to induce protein dimerization represents a promising approach to controlling over biological processes (Zhang et al., 2005; Affandi and McEvoy, 2019).

### Nucleic Acid-Induced Dimerization of Protein

**FIGURE 4:**
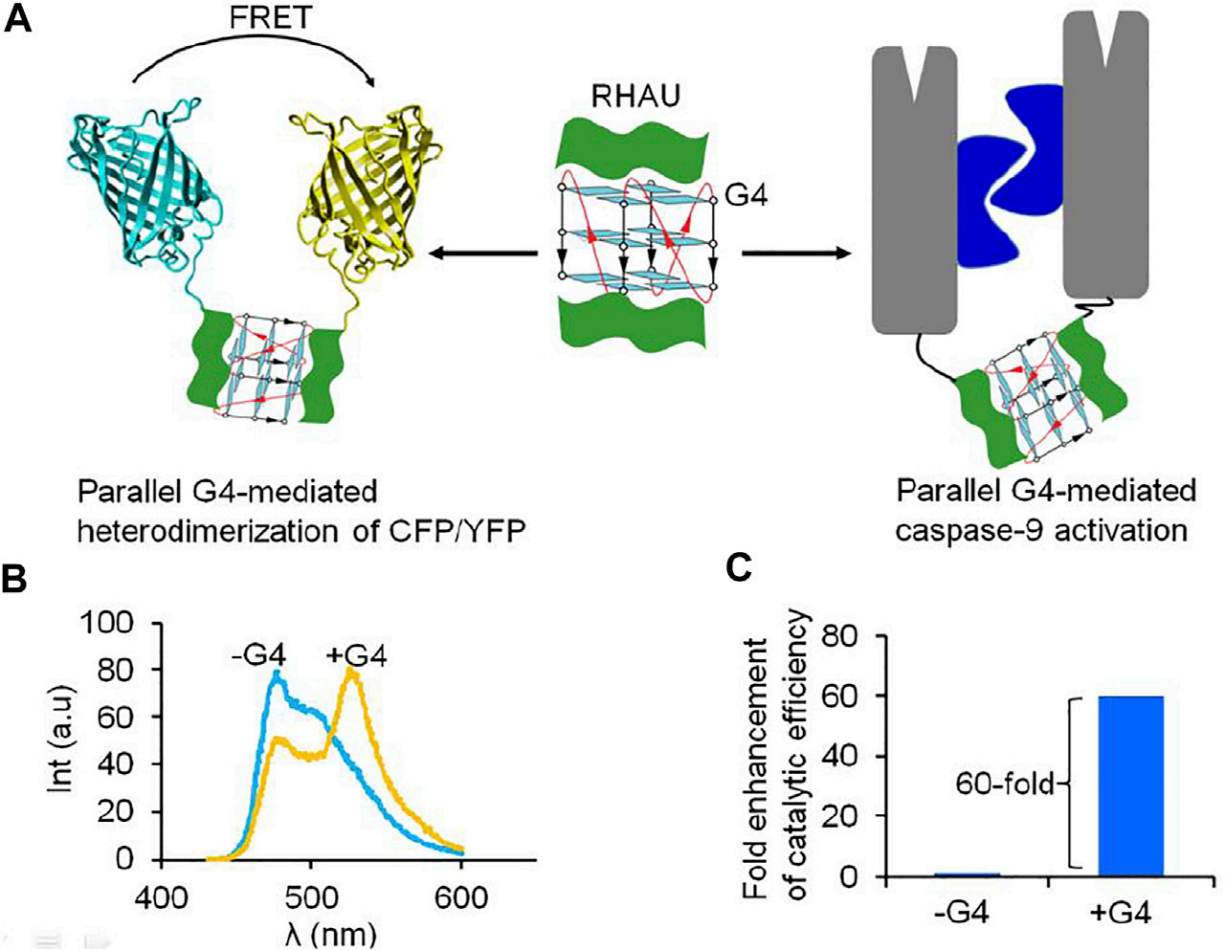
Parallel G4 can act as a target-inducer of dimerization and activation of proteins fusing with RHAU peptide. **(A)** Schematic representation of dimerization of protein and activation *via* G4, **(B)** FRET signal was observe under addition of G4 to a mixture of RHAU-CFP/RHAU-YFP (Truong et al., 2020); **(C)** G4 can play as a target molecule for inducing both dimerization and rearrangement of the active site of RHAU-caspase-9, resulting in enhancement of catalytic efficiency of enzyme (Truong et al., 2020).

G-quadruplex (G4)-induced protein dimerization has been recently reported (Truong et al., 2020). G4s are four-stranded structures formed by stacking of multiple G-tetrads. In cellular events, the formation of G4 involves in many biological processes such as replication, transcription, translation and telomeric maintenance (Lipps and Rhodes, 2009; Maizels and Gray, 2013). Therefore, specific interaction between G4 with proteins has emerged as a promising approach for regulation of biological processes. A G4-binding protein domain was also identified in N-terminus of RHAU (RHA helicase associated with AU rich element) (Heddi et al., 2015; Dang and Phan, 2019; Dang et al., 2021). Interestingly, NMR solution structure of a complex of an 18-residue peptide (RHAU18) consisting a G4-specifice binding domain and a parallel G4 has showed G4 molecule can simultaneously binds two RHAU peptides at the 3′ and 5′ end G-tetrads (Heddi et al., 2015). The helical RHAU peptides covers and clamps the G4 with three-anchor-point electrostatic interactions between negatively charged phosphate groups of the G4 and the three positively charged amino acids (K_8_, R_10_, K_19_) of the peptide. The specific binding of parallel G4 to two RHAU peptide provides a promising approach for G4-induced self assembly of protein by fusing a functional protein with RHAU peptide (Heddi et al., 2015). Incorporating a RHAU peptide with a fluorescent protein pair: cyan fluorescent protein/yellow fluorescent protein (CFP/YFP), resulting in generation of a pair of FRET (fluorescence resonance energy transfer) RHAU-CFP/RHAU-YFP. Upon addition of G4 to a mixture of RHAU-CFP and RHAU-YFP, the energy transfer from the donor CFP to the acceptor YFP was observed by G4-induced heterodimerization of proteins (Truong et al., 2020) (Figure 4). In addition, G4-induced dimerization protein approach was applied for dimer-driven activation of caspase-9. Inactivated monomeric caspase-9 incorporating with RHAU peptide allowed G4 to regenerate a catalytic activity. In the presence of G4, the catalytic efficiency of caspase-9 was 60-fold enhancement towards the natural substrate. G4 can play as a target molecule for inducing both dimerization and rearrangement of the active site of caspase-9. Another study showed oligonucleotides containing (GT) repeats could induce dimerization of HIV-1 Gag protein (Zhao et al., 2019). Short oligonucleotide as (GT)_3_ or (GT)_8_ bound to nucleocapsid (NC) domain of Gag protein leading to change conformation of Gag that is favor for Gag dimerization. Induction of dimerization of protein by nucleic acid is an alternative approach to study on function of protein and interplay between protein dimerization state and activation, not only enzyme, but also many other protein homodimerization events.

### Supramolecular System-Induced Dimerization of Protein

**FIGURE 5:**
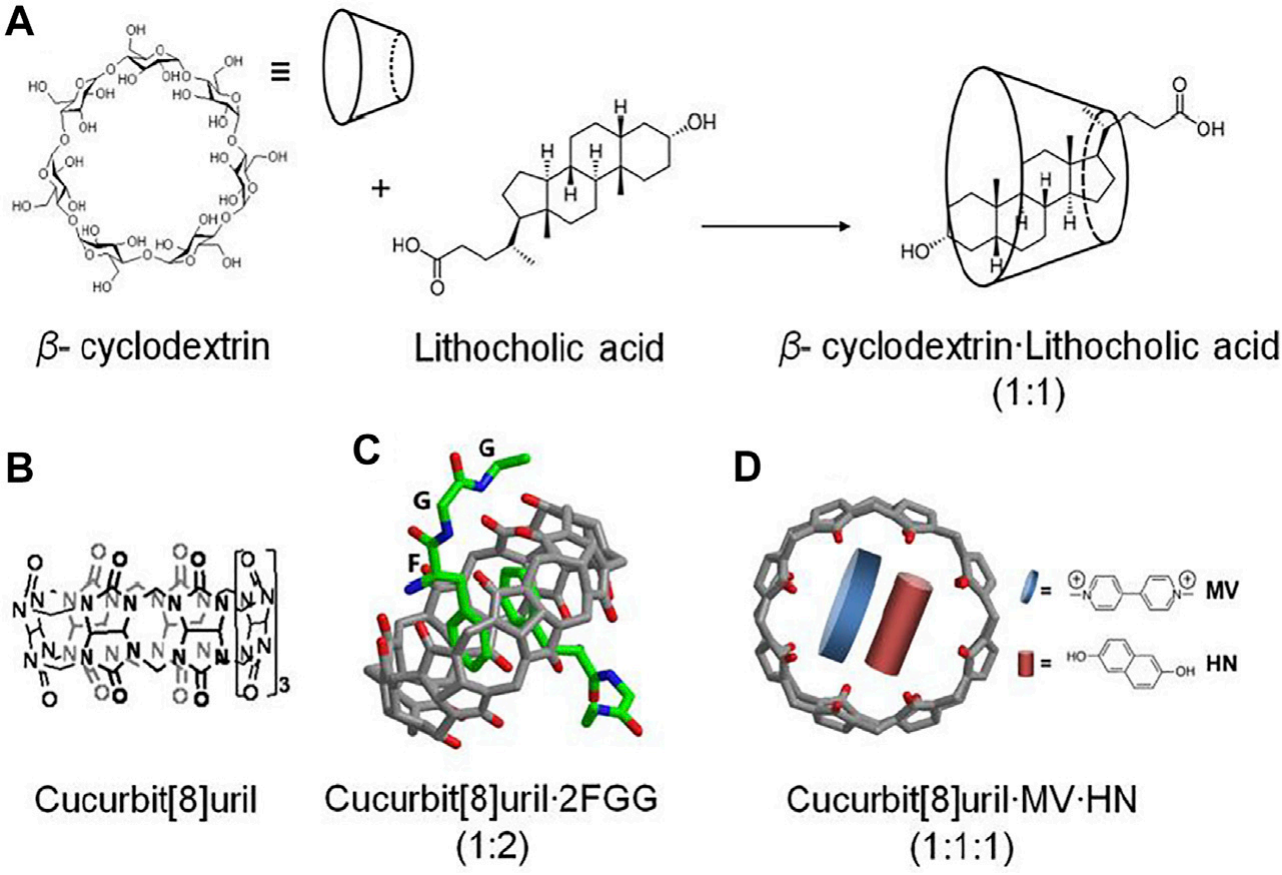
**(A)** β-cyclodextrin recognizes and binds lithocholic acid forming a complex (1:1) (Yang and Breslow, 1997), **(B)** Chemical structure of cucurbit[8]uril, **(C)** cucurbit[8]uril selectively binds and dimerizes tripeptide phenylalanineglycine·glycine (FGG) (Heitmann et al., 2006), **(D)** ternary complex of cucurbit[8]urilmethylviologen·dihydroxynapthalene (Q8:MV:HN) (Ko et al., 2007; Rauwald et al., 2010).

Supramolecular chemistry was initially inspired by biomolecules and their higher order structures (Uhlenheuer et al., 2010; Khan and Lee, 2021). Recently, the supramolecular chemistry to modulate and control dimerization of protein have been reported. Supramolecular systems bearing natural or synthetic components have been engineered with desirable properties for use in biochemical research, such as improved water solubility and guest-specific binding (Oshovsky et al., 2007). The application of supramolecular chemistry for protein dimerization is based on the non-covalent interaction of supramolecular hosts with specific guest molecules, while being appended to proteins. Two supramolecular host molecules, cyclodextrin and cucurbit [8]uril, have been most intensively explored as tools for the selective and reversible control over protein dimerization in both buffered solution and living cells (Zhang et al., 2007; Nguyen et al., 2010). Cyclodextrins are naturally-derived sugar-based cone-shaped host molecules, which selectively bind hydrophobic guest molecules to form, typically, a 1:1 complex in aqueous solution. For example, the cavity of a *β*-cyclodextrin variant recognizes and binds lithocholic acid with high affinity (*K*_a_ = 10^6^ M^−1^) (Yang and Breslow, 1997) (Figure 5A), opening up the possibility for *β*-cyclodextrin to recognize and bind protein-lithocholic acid conjugates. Cucurbit [8]uril is the eight membered homologue of the cucurbit [n]uril family of glycoluril based macrocycles, which has shown highly attractive biochemical applications due to its capacity to bind various cationic guest molecules, in addition to its good water-solubility and low toxicity (Urbach and Ramalingam, 2011; Masson et al., 2012). The cavity of cucurbit [8]uril is sufficiently large to bind two synthetic guest molecules simultaneously such as two equivalents of *N*-phenylpiperazine, aminoacridiziniums, naphthyl derivatives, coumarin and neutral red under acidic conditions (Urbach and Ramalingam, 2011) (Figures 5B–D). The favorable recognition of two guests by cucurbit [8]uril enables the formation of highly stable ternary complexes in aqueous solution. The selective non-covalent interaction of cucurbit [8]uril and guest elements provides a means to reversibly control dimerization of proteins incorporating these supramolecular guest elements. The easy design and synthesis of supramolecular host-guest systems opens up the possibility of modulating and controlling dimerization of protein.

**FIGURE 6:**
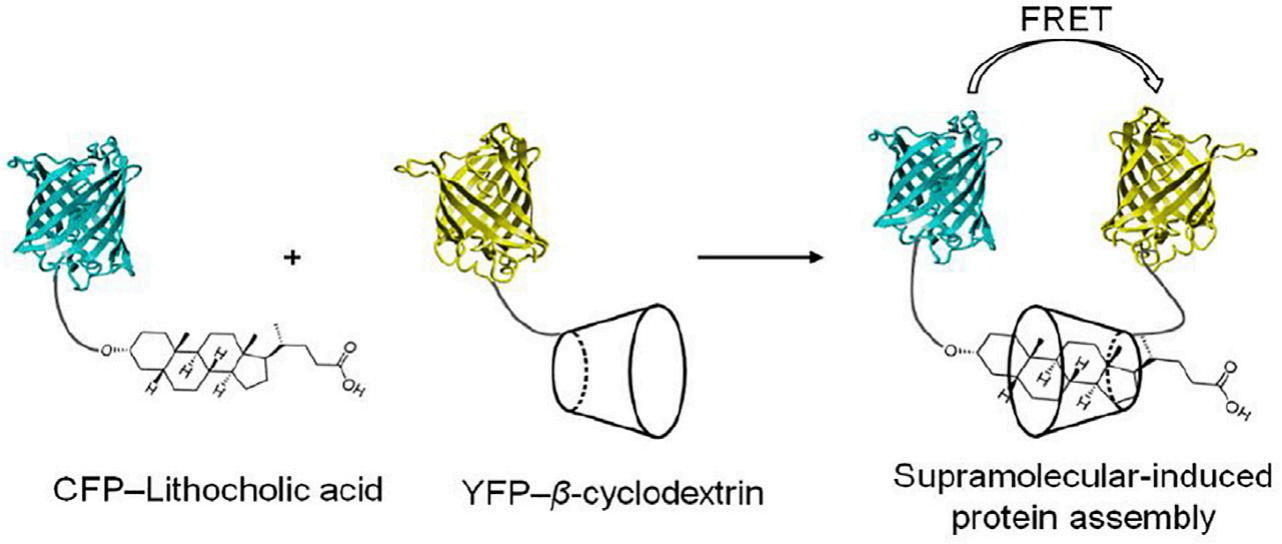
Schematic representation of cyclodextrin-induced assembly of CFP and YFP functionalized with lithocholic acid and cyclodextrin, respectively (Zhang et al., 2007).

The concept of supramolecular induced protein-protein interactions was first probed using supramolecular host-guest elements attached to synthetic peptides (Ueno et al., 1993). The specific recognition of the adamantyl group by *β-*cyclodextrin permitted a *β-*cyclodextrin-conjugated synthetic peptide to selectively recognize and bind an adamantane-conjugated peptide. This self-assembled peptide dimer enabled strong and selective DNA recognition. DNA recognition by supramolecular peptide dimerization could be reversed by inhibiting the supramolecular dimerization with either free *β-*cyclodextrin or adamantane. The fluorescent proteins were conjugated to the *β-*cyclodextrin and lithocholic acid host guest system at the C-terminus of the proteins (Zhang et al., 2007) (Figure 6). A high affinity and selective recognition of lithocholic acid by β-cyclodextrin enabled the association of the two fluorescent proteins. In this case, the degree of protein association could be monitored by donor-receptor fluorescence resonance energy transfer (FRET), both in buffer and in cells. Increasing the affinity of the synthetic host-guest complex would enhance the interaction of host-guest conjugated proteins and would thus be attractive from the point of view of studying protein-protein interactions at lower concentrations. This concept could be shown using a *β-*cyclodextrin host which was modified to heptakis-[6-deoxy-6-(2-aminoethyl-sulfanyl]-*β-*cyclodextrin (Gomez-Biagi et al., 2008). This molecular upgrading of the *β-*cyclodextrin side-chains brought about a 10 fold increase in binding to lithocholic acid and enhanced FRET (Uhlenheuer et al., 2011a). The optimization of synthetic host-guest systems is not a unique approach to increase the affinity of protein-protein interactions: engineering of the dimeric interface of the proteins can also been used to increase and stabilize the supramolecular protein dimerization. For example, point-mutated (S208F and V224L) fluorescent CFP and YFP proteins, so called dimerizing proteins (dCFP and dYFP) which normally show weak intrinsic affinity for dimerization, formed strong and stable supramolecular protein complexes on ligation of host-guest elements with a concomitant very strong FRET (Uhlenheuer et al., 2009). The reversibility of the supramolecular protein dimerization, could be probed by addition of *β-*cyclodextrin to the supramolecular protein dimers, resulting in inhibition of protein dimerization for all types of protein pairs (Zhang et al., 2007; Uhlenheuer et al., 2009; Uhlenheuer et al., 2010). *β*-Cyclodextrin can also be used to induce homodimerization of proteins (Kitagishi et al., 2012). For example, surface functionalization of bovine serum albumin protein (BSA) with TMe- *β*-cyclodextrin enabled 5,10,15,20-tetrakis (4-sulfonatophenyl)porphyrin to reversibly control BSA homodimerization via complexation with the TMe- *β*-cyclodextrin. The resulting supramolecular protein dimer is stable and can be separated from monomeric proteins via size exclusion chromatography.

Cucurbit [8]uril is a second attractive supramolecular host molecule for use in reversible protein dimerization studies (Bai et al., 2016; Cao et al., 2021; Liu et al., 2021). Cucurbit [8]uril selectively binds and dimerizes two guest molecules simultaneously within its hydrophobic cavity, and with high affinity (Heitmann et al., 2006; Ko et al., 2007). For example, cucurbit [8]uril recognizes and binds methyl viologen (MV) and naphthalene (Np) simultaneously to form a ternary cucurbit [8]urilMV·Np complex (Ko et al., 2007). This concept could be applied to induce heterodimerization of CFP and YFP. For this, CFP and YFP were chemically outfitted with Np and MV, resulting in synthetic CFP-Np and YFP-MV mutants, respectively. The addition of cucurbit [8]uril mediated heterodimerization of CFP-Np with YFP-MV, resulting in an energy transfer from donor CFP to acceptor YFP (Uhlenheuer et al., 2011b). Incorporation of a 4,4′-bipyridinium scaffold at the C-teminus of transcription factor (GCN4) opened up a new approach to the supramolecular control of peptide assemblies (Novo et al., 2021). Cucurbit [8]uril-induced GCN4 dimerization by the formation of a homoternary supramolecular complex (1 cucurbit[8]uril:2 bipyridinium) could specifically bind to its targeted double-strand DNA. This binding complex was easily disassembled in a reversible manner upon addition of a specific competitor guest. Interestingly, cucrbit[8]uril-induced dimerization of antimitotic peptide-conjugated benzylimidazolium could recognize the microtubules and convert from fibrous to nanoparticulate aggregates through cross-linkage of host-guest complex. The cucurbit[8]uril-induced intertubular aggregation was applied to regulate cell apoptosis and tumor ablation at the cellular level and in the mouse (Zhang et al., 2019). Chemical conjugation of specific guest molecules with proteins or peptides provides a facile supramolecular method to enhance protein-protein interactions which may open up new opportunities for biomedical applications.

**FIGURE 7:**
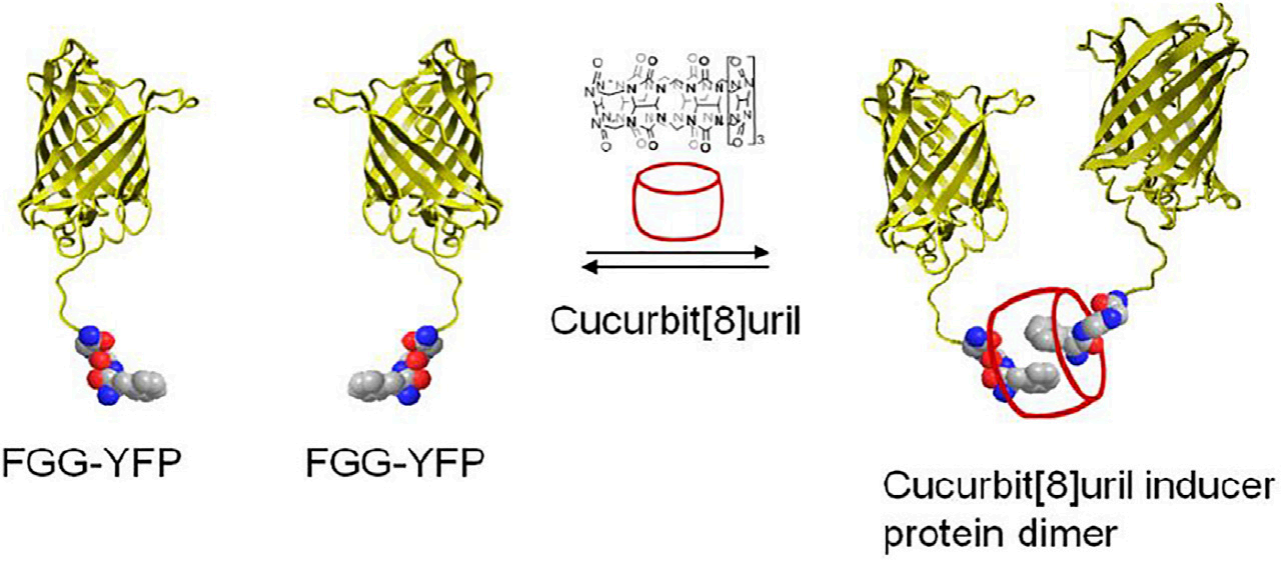
Schematic representation of two yellow fluorescent proteins having an N-terminal FGG peptide motif and their dimerization which is induced by cucurbit[8]uril (Nguyen et al., 2010).

The supramolecular host cucurbit[8]uril can as well be efficiently used to reversibly switch the dimerization of fluorescent proteins incorporating a genetically encoded N-terminal phenylalanine-glycine-glycine (FGG) peptide motif (Nguyen et al., 2010; Dang et al., 2014) (Figure 7). The proteins with an FGG-tag are easily generated by autocleavage of an intein system under control of pH and temperature. Selective binding of the FGG-tag to the hydrophobic cavity of cucurbit[8]uril induces protein dimerization and is mediated via a key interaction between the N-terminal amine functionality of the peptide and the carbonyl rim of cucurbit [8]uril (Figure 5C), resulting in protein homo- or heterodimerization. Cucurbit[8]uril-induced dimerization of proteins bearing an FGG-tag via a supramolecular host-guest interaction can be fully reversed through the addition of a small synthetic competitor (methyl viologen) (Nguyen et al., 2010). In addition, cucurbit[8]uril has been used as an inducer of protein tetramerization (dimer of dimer), by combining the two-fold binding of an FGG motif to cucurbit[8]uril with intrinsic affinities between the proteins domains as a stepwise assembly process (Dang et al., 2012). The incorporation of a dimerizing interface at the fluorescent protein surface (dYFP, dCFP) combined with an encoded N-terminal phenylalanine-glycine-glycine (FGG) peptide motif allowed cucurbit[8]uril to selectively recognize and induce FGG-dYFP or FGG-dCFP homotetramerization. The concept of cucurbit[8]uril-induced protein homotetramerization was elucidated using a combination of dynamic light scattering and size exclusion chromatography experiments. Addition of cucurbit[8]uril to a solution of FGG-dYFP, pre-dimerized in solution, resulted in the automatic generation of the tetrameric protein assembly.

**FIGURE 8:**
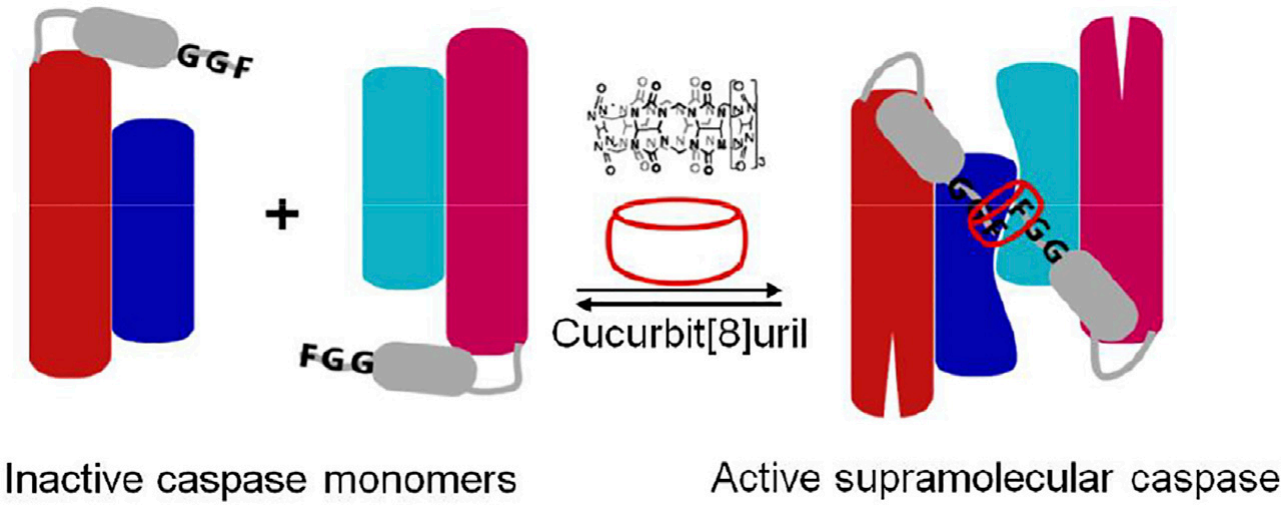
Schematic representation of N-terminal FGG-bearing (grey) monomeric caspase-9 (red: large subunit, blue: small subunit) and its dimerization into an enzymatically active homodimer by cucurbit[8]uril (Dang et al., 2013).

Cucurbit[8]uril-induced self assembly of protein dimerization approach has been applied to study molecular mechanisms of dimerization and activation of caspase-9 (Dang et al., 2013) (Figure 8). Caspase-9 consisting of FGG motif (FGG-caspase-9) at the N-terminus allows cucurbit[8]uril to induce protein dimerization which was confirmed by dynamic light scattering (DLS). The catalytic activity of enzyme increases upon increased addition of cucurbit[8]uril until a maximal activity is reached when all FGG-caspase-9 is dimerized. The activity of the cucurbit[8]uril-induced FGG-caspase-9 dimer is not only significantly greater than that of the isolated protein, but is also superior to proteins mutated to have an engineered hydrophobic dimerization interface. Upon addition of a competitor peptide (FGG) to the active cucurbit[8]uril-induced FGG-caspase-9 dimer, the enzymatic activity of enzyme was decreased in a dose-dependent fashion (Dang et al., 2013). The reversibility of the cucurbit [8]uril–FGG system thus shows the full control achievable over dimerization of FGG-caspase-9 dimerization and activation via supramolecular host–guest approach and the potential to either induce or inhibit protein dimerization with specific small guest molecules. Moreover, light-triggered supramolecular cucurbit[8]uril activation of FGG-caspase-9 has been demonstrated (de Vink et al., 2021). Cucurbit [8]uril was temporarily caged by bivalent FGG peptide with high affinity. The UV light induced release of cucurbit[8]uril from a bivalent cage molecule, resulting in activation of cucurbit[8]uril-induced FGG-caspase-9 dimerization. The concept of light-responsive caged cucurbit[8]uril also provides a new platform for application of switchable approaches. Supramolecular reactivation of inactivated enzymes have been studied on inactivated caspase-8 mutant and split-luciferase fragments. A mutated caspase-8 (D384A) featuring FGG motif at the N-terminus which shows enzymatically inactive towards its natural substrate caspase-3, could be fully reactivated upon addition of cucurbit[8]uril (Dang et al., 2018). The FGG motif was applied to split-luciferase fragment pairs at the N-terminus that allowed cucurbit[8]uril to induce dimerization of luciferase and regenerate enzymatic activity (Bosmans et al., 2016). Cucurbit[8]uril can act as a supramolecular inducer of dimerization, thus leading to optimal protein reorganization and enzymatic activation that holds great promises for studying many other protein homodimerization events in a reversible manner, such as dimerizing enzymes and membrane receptor proteins.

**FIGURE 9:**
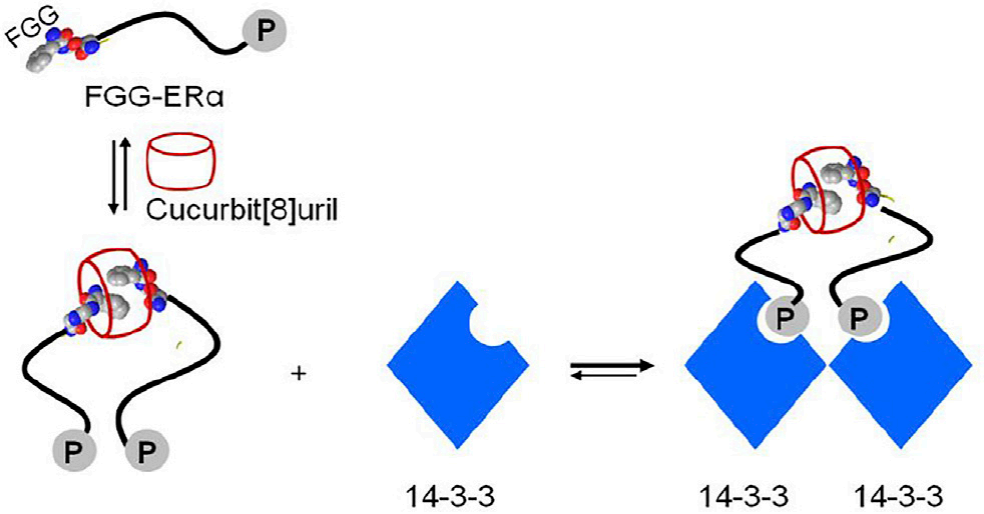
Schematic representation of cucurbit[8]uril-induced protein assemblies of phosphopeptide (FGG-ERα) and protein 14-3-3.

Crystal structure of the supramolecular-mediated protein complex has been studied on cucurbit[8]uril-induced dimerization of protein 14-3-3 (involved in human disease including the breast-cancer target) (de Vink et al., 2017; Liu et al., 2021). Fusion of FGG motif to the N-terminus of the 14-3-3 binding epitope of the estrogen receptor alpha (ERα) could be simply formed into a dimeric peptide in the presence of cucurbit[8]uril. Cucurbit[8]uril-induced ERα peptide dimerization significantly enhanced its affinity towards protein 14-3-3 *via* a binary bivalent binding manner (Figure 9). Molecular insight into the supramolecular interaction of the complex of protein, peptide and cucurbit[8]uril was clarified by the first crystal structure (de Vink et al., 2017). The crystal structure showed that the complex was favorably stabilized by multiple intermolecular interactions. The cucurbit[8]. FGG system has also been applied to generate protein nanowires (Hou et al., 2013; Bai et al., 2016). Genetic generation of the dimeric glutathione S-transferase (GST) surfaces consisting of FGG motif at the symmetric N-terminus allowed cucurbit[8]uril to induce self-assembly of protein into high molecular nanowires (Hou et al., 2013). The incorporation of Se-containing active center to FGG-GST resulted in a functionalized Se-FGG-GST. The Se-FGG-GST could be easily formed into high molecular nanowires in the presence of cucurbit[8]uril which was shown to be a better antioxidant than Se-FGG-GST monomers. That holds a great promise for the design of functional proteins such as biosensors, catalysis and pharmaceuticals.

Approaches of controlling protein dimerization reveals diversity in the regulation of protein activity both *in vitro* and *in vivo*. It is necessary to apply these approaches to therapeutic applications. Some molecule glues-induced protein dimerization have been used as drugs in clinical treatment. For example, rapamycin and its analog have been approved by FDA (Food and Drug Administration) as an immunosuppressive drug for transplantation and cancer therapy (Schreiber, 2021). Interestingly, ARV-110 is the first “PROTAC” molecule glue which entered phase I clinical trials (Schreiber, 2021). In addition, supramolecular system-induced protein dimerization also shows great potential for therapeutic applications. The administration of cucurbit [8]uril-induced aggregation of tubulin-targeted antimitotic peptides could induce apoptosis and suppress tumor growth which can be developed as a therapeutic supramolecular approach for cancer treatment (Zhang et al., 2019). The host-guest cucurbit [8]uril:FGG (1:2) complex has been widely used to regulate numerous functional proteins such as caspase-9, caspase-8, protein 14-3-3, nanowires that also holds a great promise for design of functional proteins such as biosensors, catalysis and pharmaceuticals (Liu et al., 2021).

Protein dimerization plays a key role in almost all biological processes. Control over protein dimerization using molecules is an important concept for studying the fundamental underlying molecular processes. The use of molecules to induce protein dimerization in part overcomes the limitations of protein engineering approaches. In particular, the selective recognition of small guests by synthetic host molecules to form 1:1 complexes or 1:2 ternary complexes enables the reversible control of protein dimerization using proteins prefunctionalized with small guest elements. The supramolecular induced dimerization of protein represents orthogonal approaches for studying functional protein dimerization and aggregation, thus opening up new opportunities for biomedical applications.
